# “Omega-3 fatty acids and Alzheimer’s disease: current evidence and emerging insights”

**DOI:** 10.1097/MS9.0000000000004124

**Published:** 2025-10-17

**Authors:** Amisha Kumari, Archana Kumari, Muddassir Khalid, Supoma Ghosh Ria

**Affiliations:** a Department of Medicine, Liaquat University of Medical and Health Sciences, Jamshoro, Pakistan; b Department of Medicine, Jinnah Sindh Medical University, Karachi, Pakistan; c Department of Medicine, Nishtar Medical University, Multan, Pakistan; d Mugda Medical College, University of Dhaka, Dhaka, Bangladesh

*Dear Editor,*

Alzheimer’s disease (AD) remains a growing burden to patients, families, and health care systems, thus making it difficult to overemphasize the necessity of preventing or disease-modifying approaches. The omega-3 polyunsaturated fatty acids (PUFAs), especially docosahexaenoic acid (DHA) and eicosapentaenoic acid (EPA), have been explored as a potential neuroprotective agent due to their role in maintaining synaptic integrity, regulating anti-inflammatory mechanisms, and preserving neuronal membrane functionality.

The omega-3 fatty acids, especially DHA, have been the focus of numerous studies regarding their potential to exert neuroprotective and cognitive-preserving effects. Initial clinical trials yielded mixed results; however, according to emerging data, the therapeutic effect may depend on the timing of action and patient phenotype. Recently, a randomized, double-masked, and placebo-controlled trial conducted in 2025 demonstrated that 12-month supplementation with medium-chain triglyceride and DHA improves cognitive performance in individuals with mild cognitive impairment^[1]^. Another 2023 network meta-analysis of 23 randomized controlled trials (RCTs) in patients with AD found that EPA-based omega-3 preparations with antioxidants were most likely to have a cognitive effect, and DHA-only preparations provided relatively weaker effects^[2]^.

The relation is further elucidated by randomized trials carried out on high-risk cohorts. A 2021 RCT in Japanese participants aged 65 years found that 1200 mg DHA daily reduced hippocampal atrophy and maintained memory performance after 18 months compared to placebo^[3]^. Similarly, a 2024 two-blind, randomized trial aimed at APOE-4 carriers found that high-dose omega-3 supplementation maintained neuronal integrity on magnetic resonance imaging and slowed the rate of white-matter lesion development in this genetic risk group^[4]^. In a 2025 network meta-analysis of RCTs in patients with Alzheimer’s dementia, long-term high-dose (1500–2000 mg/day) EPA-dominant omega-3 PUFAs with antioxidants demonstrated the highest potential for cognitive benefit among all treatments evaluated^[5]^.

In conclusion, recent RCTs and meta-analyses suggest that the protective effects of omega-3 fatty acids are not consistent; on the contrary, they seem to be most effective in early disease progression or in high-risk groups, including carriers of APOE 4. Subsequently, the large-scale trials that will be implemented in the future must be stratified based on genotype and directed, with the help of biomarker data, to define the optimal dosing, formulation, and timing of administering the drugs.

Omega-3 supplementation is currently a safe and biologically plausible intervention, but it needs to be repositioned as an adjunctive preventive measure, rather than a disease-modifying treatment.

TITAN Guidelines: This manuscript complies with TITAN Guidelines, 2025, declaring no use of artificial intelligence^[6]^.

## Data Availability

Not applicable.
